# Effects of once-weekly semaglutide 2.4 mg on C-reactive protein in adults with overweight or obesity (STEP 1, 2, and 3): Exploratory analyses of three randomised, double-blind, placebo-controlled, phase 3 trials

**DOI:** 10.1016/j.eclinm.2022.101737

**Published:** 2022-11-29

**Authors:** Subodh Verma, Meena Bhatta, Melanie Davies, John E. Deanfield, W. Timothy Garvey, Camilla Jensen, Kristian Kandler, Robert F. Kushner, Domenica M. Rubino, Mikhail N. Kosiborod

**Affiliations:** a Division of Cardiac Surgery, St Michael's Hospital, University of Toronto, Toronto, ON, Canada; b Novo Nordisk, Søborg, Denmark; c Diabetes Research Centre, University of Leicester, Leicester, UK; d NIHR Leicester Biomedical Research Centre, Leicester, UK; e Institute of Cardiovascular Science, University College London, London, UK; f Department of Nutrition Sciences, University of Alabama at Birmingham, Birmingham, AL, USA; g Division of Endocrinology, Feinberg School of Medicine, Northwestern University, Chicago, IL, USA; h Washington Center for Weight Management and Research, Arlington, VA, USA; i Department of Cardiovascular Disease, Saint Luke's Mid America Heart Institute and University of Missouri-Kansas City School of Medicine, Kansas City, MO, USA

**Keywords:** C-reactive protein, Cardiovascular risk factors, Semaglutide, Obesity, Type 2 diabetes, Weight loss, Inflammation, Weight management, GLP-1RA

## Abstract

**Background:**

Inflammation is a key driver of atherosclerotic cardiovascular disease. C-reactive protein (CRP), an established biomarker of inflammation, is commonly elevated in people with overweight/obesity.

**Methods:**

STEP 1, 2, and 3 were 68-week, placebo-controlled trials of semaglutide for weight management in participants with overweight/obesity, with (STEP 2) or without (STEP 1 and 3) type 2 diabetes. Change in serum CRP from baseline to week 68 was assessed as a prespecified secondary endpoint for semaglutide 2.4 mg versus placebo (STEP 1, 2, and 3) and versus semaglutide 1.0 mg (STEP 2). *Post hoc* assessments included change in CRP by baseline characteristics (bodyweight, body mass index [BMI], glycaemic status, CRP concentration); change in CRP-defined cardiovascular risk category (<1 [low], 1–3 [intermediate], and >3 mg/L [high]); and correlation between change in CRP and change in bodyweight, waist circumference, fasting serum insulin (STEP 1 and 3), fasting plasma glucose, and homeostatic model assessment of insulin resistance (HOMA-IR).

**Findings:**

The trials took place from June through November 2018 (STEP 1 and 2) and from August 2018 to April 2020 (STEP 3). In all trials, semaglutide 2.4 mg reduced CRP at week 68 versus placebo (estimated treatment difference [ETD; 95% CI] −44% [–49 to −39] in STEP 1, –39% [–46 to −30] in STEP 2, and –48% [–55 to −39] in STEP 3; all p < 0.05). In STEP 2, CRP reductions were greater with semaglutide 2.4 mg (−49%) than with 1.0 mg (−42%) but the difference did not reach statistical significance (ETD [95% CI] −12% [–23 to 1]; p = 0.06). Reductions in CRP occurred in parallel with bodyweight loss and were consistent regardless of baseline BMI/bodyweight/glycaemic status. More semaglutide-treated participants had reductions in CRP-defined cardiovascular risk versus those on placebo. Reductions in CRP were positively correlated with reductions in bodyweight, waist circumference, fasting plasma glucose, fasting serum insulin, and HOMA-IR (data not shown).

**Interpretation:**

In people with overweight/obesity, once-weekly semaglutide 2.4 mg and 1.0 mg reduced CRP concentration irrespective of baseline BMI/bodyweight/glycaemic status compared with placebo. These data suggest a potential anti-inflammatory role of semaglutide in obesity.

**Funding:**

10.13039/501100004191Novo Nordisk.

**Research in context**

**Evidence before this study**

Obesity is associated with systemic inflammation, as reflected by elevated levels of C-reactive protein (CRP). The relationship between systemic inflammation and development of cardiovascular disease is well documented. Weight loss is associated with reductions in CRP and hence inflammation. We searched PubMed on 28 June 2021 for articles published in the past 5 years, with no language restrictions, using the search terms “cardiometabolic”, “cardiovascular”, “C-reactive protein”, “inflamm∗”, “glucagon-like peptide-1 receptor agonist”, “obesity”, and “overweight”.

Glucagon-like peptide-1 (GLP-1) receptor agonists (GLP-1RAs) promote weight loss and have demonstrated anti-inflammatory effects, including reductions in CRP. A meta-analysis of prospective cohort studies and randomised controlled trials ranging from 8 to 52 weeks demonstrated that GLP-1RA treatment was associated with significant reductions in CRP concentrations in people with type 2 diabetes, with the duration of treatment associated with degree of CRP reduction.

Semaglutide is a GLP-1 analogue that reduces CRP concentrations and reduces cardiovascular risk in people with type 2 diabetes.

**Added value of this study**

Semaglutide at a higher dose of 2.4 mg significantly reduced CRP concentrations compared with placebo in people with overweight or obesity with or without type 2 diabetes, regardless of baseline body mass index, bodyweight, or glycaemic status. Reductions in CRP occurred in parallel with bodyweight loss and were positively correlated with reductions in bodyweight, waist circumference, fasting plasma glucose, fasting serum insulin, and homeostatic model assessment of insulin resistance.

**Implications of all the available evidence**

Using CRP as a surrogate marker, semaglutide 2.4 mg reduces inflammation and may therefore reduce cardiovascular risk in people with overweight or obesity. The ongoing Semaglutide Effects on Heart Disease and Stroke in Patients with Overweight or Obesity (SELECT) trial is investigating whether semaglutide 2.4 mg is superior to placebo for preventing major adverse cardiovascular events in participants with established cardiovascular disease and overweight or obesity, but without diabetes.

## Introduction

Obesity can exacerbate insulin resistance, which further worsens low-grade chronic inflammation, dyslipidaemia, and hypertension, thus placing people with obesity at increased risk of developing cardiovascular disease and type 2 diabetes.^1^ C-reactive protein (CRP) is an acute inflammatory protein that reflects the systemic inflammation that is a component of the insulin-resistant state. CRP is elevated in obesity and is associated with the development of cardiovascular disease.^2^, ^3^, ^4^ Consequently, CRP has sometimes been used as a biomarker of cardiovascular risk. CRP concentrations of <1 mg/L, 1–3 mg/L, and >3 mg/L indicate low, intermediate, and high relative cardiovascular risk, in the context of traditional risk factors.^2^^,^^3^ Elevation of CRP is a downstream effect of pro-inflammatory signalling by interleukin-6, which is itself triggered through the activation of interleukin-1 by the NLRP3 inflammasome.^5^ Trials have shown that targeted inhibition of this inflammatory pathway, as measured by changes in CRP concentrations, can reduce the rate of cardiovascular events.^5^, ^6^, ^7^ Losing weight is associated with reductions in CRP and therefore inflammation, regardless of the modality used to promote weight loss.^2^^,^^8^

Glucagon-like peptide-1 (GLP-1) receptor agonists (GLP-1RAs) are known to improve glycaemic control and reduce bodyweight. They have also been shown to have anti-inflammatory effects, including reducing CRP.^9^^,^^10^ Semaglutide is a GLP-1 analogue that is available as a once-weekly subcutaneous (s.c.; at doses up to 2.0 mg) and a once-daily oral (at doses up to 14 mg) formulation for the treatment of type 2 diabetes.^11^^,^^12^ The efficacy and safety of s.c. semaglutide at a greater dose of 2.4 mg once weekly has been investigated for weight management in people with overweight or obesity, with or without weight-related complications, in global phase 3a Semaglutide Treatment Effect in People with Obesity (STEP) trials. In the first of the STEP trials (STEP 1–4), 68 weeks of once-weekly s.c. semaglutide 2.4 mg treatment resulted in mean weight loss of 15%–17% in adults with overweight or obesity and 10% in those who also had type 2 diabetes.^13^, ^14^, ^15^, ^16^ Semaglutide 2.4 mg is now approved in several countries for weight management in adults with obesity or with overweight and at least one weight-related comorbidity.^17^, ^18^, ^19^

Changes in serum CRP concentrations were assessed for semaglutide 2.4 mg versus placebo in STEP 1, 2, and 3 and versus semaglutide 1.0 mg in STEP 2. Here we report the effects of semaglutide on CRP across all three trials, examining these effects across subgroups of baseline bodyweight, glycaemic control, and CRP. We also evaluate the association of changes in CRP with changes in bodyweight and other selected cardiometabolic outcomes.

## Methods

Full details of the methods used in STEP 1, 2, and 3 have previously been published.^13^, ^14^, ^15^

### Trial designs and participants

Briefly, STEP 1, 2, and 3 were all 68-week, randomised, double-blind, placebo-controlled trials of semaglutide 2.4 mg (STEP 1–3) and 1.0 mg (STEP 2), with safety and tolerability assessed up to week 75. All trials complied with the International Conference on Harmonisation Good Clinical Practice guidelines and the Declaration of Helsinki. The protocols and amendments were approved by the relevant institutional review board or independent ethics committee at each trial site. All participants provided written consent to take part in the trials.^13^, ^14^, ^15^

Adults (18 years or older) were enrolled. In STEP 1 and 3, participants were required to have a body mass index (BMI) of either at least 30 kg/m^2^ or at least 27 kg/m^2^ combined with at least one weight-related comorbidity (but not type 2 diabetes).^14^^,^^15^ In STEP 2, participants were required to have a BMI of at least 27 kg/m^2^, glycated haemoglobin (HbA_1c_) of 7%–10%, and a diagnosis of type 2 diabetes that was managed by diet and exercise alone or with up to three oral glucose-lowering therapies.^13^

### Randomisation and masking

In all three trials, participants were randomly assigned to treatment. STEP 1 and 3 used a double-blind design and participants were randomised 2:1 to either once-weekly s.c. semaglutide 2.4 mg or placebo.^14^^,^^15^ In STEP 2, participants were randomised 1:1:1 to once-weekly s.c. semaglutide 2.4 mg, semaglutide 1.0 mg, or placebo, with randomisation stratified according to background diabetes treatment and HbA_1c_ (above or below 8.5%). STEP 2 used a double-blind, double-dummy design.^13^

Participants and trial site staff were blinded to treatment allocation, as were those analysing the data until masking was broken at database lock.

### Procedures

Semaglutide was initiated at 0.25 mg per week and escalated in a fixed-dose regimen every 4 weeks until the target dose was achieved (2.4 mg by week 16, or 1.0 mg by week 8 [STEP 2 only]). In STEP 1 and 2, trial product was given as an adjunct to lifestyle interventions (counselling on diet and exercise); in STEP 3 it was given as an adjunct to intensive behavioural therapy (decreased energy intake, increased physical activity, and counselling sessions).

### Outcomes

All outcomes were assessed as changes from baseline to week 68 for each treatment group in each trial. The overall change in CRP concentration by treatment was assessed as a prespecified secondary endpoint in each trial. All other outcomes were *post hoc*.

Subgroup analyses were performed to assess the effect of participant characteristics on the change in CRP concentration in each treatment group. For these analyses, participants were stratified into subgroups of the following clinical characteristics at baseline: bodyweight (<90, 90–<100, 100–<115, and ≥115 kg), BMI (<30, 30–<35, 35–<40, and ≥40 kg/m^2^), glycaemic status (normoglycaemia or prediabetes) in STEP 1 and 3 only, and CRP concentration (using high-sensitivity CRP tests and based on the established cut-offs used for evaluating future cardiovascular risk; <1 [low], 1–3 [intermediate], and >3 mg/L [high]).^2^^,^^3^

To investigate the effect of treatment on CRP-defined cardiovascular risk category, participants were separated into subgroups using the CRP cut-offs outlined earlier. The proportions of participants who changed category were then assessed.

Correlations between the change in CRP and changes in bodyweight, waist circumference, fasting serum insulin (for STEP 1 and 3 only), fasting plasma glucose, and homeostatic model assessment of insulin resistance (HOMA-IR) were evaluated to investigate whether changes in CRP were related to changes in other cardiometabolic outcomes in each treatment group.

### Statistical analysis

Data were analysed for each trial separately. The overall changes in CRP were estimated using an analysis of covariance with randomised treatment as a factor and baseline value as a covariate (see original publications for full details).^13^, ^14^, ^15^ For the subgroup analyses, the changes in CRP by baseline characteristics were estimated separately for each parameter using an analysis of covariance with treatment, subgroup, and the interaction between treatment and subgroup as factors, and baseline CRP concentration as a covariate. For STEP 2, the model also included the stratification groups (oral glucose-lowering treatment status and HbA_1c_ category at screening), as well as the interaction between the groups.

The proportions of participants who changed CRP-defined cardiovascular risk category were summarised descriptively. The relationships between the changes in CRP and other efficacy parameters were estimated using Pearson correlation coefficients. A correlation coefficient of 0.5 was considered moderate.

Statistical analyses were based on the treatment policy estimand, which assessed the treatment effect in all participants, regardless of trial product discontinuation or use of other anti-obesity therapies,^20^ using data from the full analysis set (all randomised participants, regardless of whether they initiated treatment). In order to achieve approximately normal distribution of data, the changes in CRP were estimated on a log scale to give ratios to baseline; to aid interpretation, ratios were converted to percent changes calculated using the formula (estimated ratio – 1) × 100. Similarly, treatment effects for changes in CRP are presented as estimated relative percent differences between treatment groups based on the estimated treatment ratios and calculated using the same formula. Missing data were imputed 1000 times from retrieved participants of the same randomised treatment and the results were combined using Rubin's rules. Statistical analyses were not adjusted for multiplicity since they were exploratory in nature and conducted *post hoc*.

The STEP 1, 2, and 3 trials are all closed and completed. The trials are registered with ClinicalTrials.gov; NCT03548935 (STEP 1), NCT03552757 (STEP 2), and NCT03611582 (STEP 3).

### Role of the funding source

The funder designed the trials, oversaw their conduct, monitored trial sites, and collected and analysed the data; investigators were responsible for trial-related medical decisions and data collection. This article was drafted with active participation of all co-authors, with medical writing and editorial support paid for by the funder.

## Results

The total numbers of randomised participants in each trial were 1961 in STEP 1, 1210 in STEP 2, and 611 in STEP 3. Baseline demographics and clinical characteristics were well balanced across treatment groups in all trials (appendix p 2).

Baseline demographics and clinical characteristics by baseline CRP subgroup are shown in Table 1. In general, across the CRP subgroups, the proportions of female participants and those who were Black or African American increased, mean age decreased, and mean bodyweight, BMI, and waist circumference increased with increasing CRP concentration for all trials.

**Table 1:** Baseline demographics and clinical characteristics by trial and by baseline CRP subgroup.

	STEP 1			STEP 2			STEP 3		
	<1 mg/L	1–3 mg/L	>3 mg/L	<1 mg/L	1–3 mg/L	>3 mg/L	<1 mg/L	1–3 mg/L	>3 mg/L
Participants, n	223	574	1155	168	361	674	50	148	405
CRP, geometric mean (CV), mg/L	0.6 (42.9)	1.9 (31.3)	7.9 (70.5)	0.6 (50.3)	1.8 (32.1)	7.5 (68.1)	0.6 (48.1)	1.8 (34.2)	7.9 (70.4)
Age, years	48 (13)	49 (13)	45 (13)	58 (10)	57 (10)	54 (11)	50 (13)	48 (13)	45 (12)
Female sex, n	104 (46.6%)	385 (67.1%)	956 (82.8%)	48 (28.6%)	147 (40.7%)	417 (61.9%)	35 (70.0%)	105 (70.9%)	349 (86.2%)
Race, n									
White	135 (60.5%)	433 (75.4%)	894 (77.6%)	86 (51.2%)	247 (68.4%)	414 (61.4%)	39 (78.0%)	111 (75.0%)	310 (76.5%)
Asian	69 (30.9%)	73 (12.7%)	119 (10.3%)	73 (43.5%)	85 (23.5%)	159 (23.6%)	1 (2.0%)	4 (2.7%)	6 (1.5%)
Black/African American	12 (5.4%)	21 (3.7%)	77 (6.7%)	5 (3.0%)	17 (4.7%)	78 (11.6%)	7 (14.0%)	27 (18.2%)	80 (19.8%)
Other^a^	7 (3.1%)	47 (8.2%)	63 (5.5%)	4 (2.4%)	12 (3.3%)	23 (3.4%)	3 (6.0%)	6 (4.1%)	9 (2.2%)
Ethnicity, n									
Not Hispanic/Latino	202 (90.6%)	500 (87.1%)	961 (83.2%)	155 (92.3%)	309 (85.6%)	585 (86.8%)	43 (86.0%)	120 (81.1%)	319 (78.8%)
Hispanic/Latino	18 (8.1%)	51 (8.9%)	164 (14.2%)	13 (7.7%)	52 (14.4%)	89 (13.2%)	7 (14.0%)	28 (18.9%)	86 (21.2%)
Other^b^	3 (1.3%)	23 (4.0%)	30 (2.6%)	0	0	0	0	0	0
Bodyweight, kg	97.2 (18.7)	100.0 (18.3)	109.6 (23.1)	92.6 (16.5)	96.1 (18.3)	103.6 (23.4)	93.8 (15.6)	101.8 (19.1)	108.9 (24.1)
BMI, kg/m^2^	33.8 (5.1)	35.7 (5.1)	39.7 (7.0)	32.0 (4.1)	33.8 (4.9)	37.6 (6.7)	33.0 (3.6)	35.9 (4.9)	39.5 (7.1)
Waist circumference, cm	108.4 (12.8)	111.5 (12.8)	117.4 (15.2)	108.1 (11.0)	111.3 (11.9)	118.0 (14.8)	103.7 (11.3)	110.5 (12.9)	115.1 (16.1)
HbA_1c_, %	5.7 (0.3)	5.7 (0.3)	5.7 (0.3)	7.9 (0.8)	8.1 (0.8)	8.2 (0.8)	5.7 (0.3)	5.7 (0.3)	5.8 (0.3)
Statin/lipid-lowering agent users, n	42 (18.8%)	134 (23.3%)	128 (11.1%)	113 (67.3%)	232 (64.3%)	311 (46.1%)	11 (22.0%)	32 (21.6%)	46 (11.4%)

Data are mean (SD), unless otherwise stated, for all trial participants with a baseline CRP assessment.

BMI, body mass index; CRP, C-reactive protein; CV, coefficient of variation; HbA_1c_, glycated haemoglobin; SD, standard deviation.

a American Indian or Alaska Native, Native Hawaiian or Other Pacific Islander, not applicable (data on race are not collected for France), or other.

b Not applicable (data on ethnicity are not collected for France) or unknown.

**Fig. 1:**
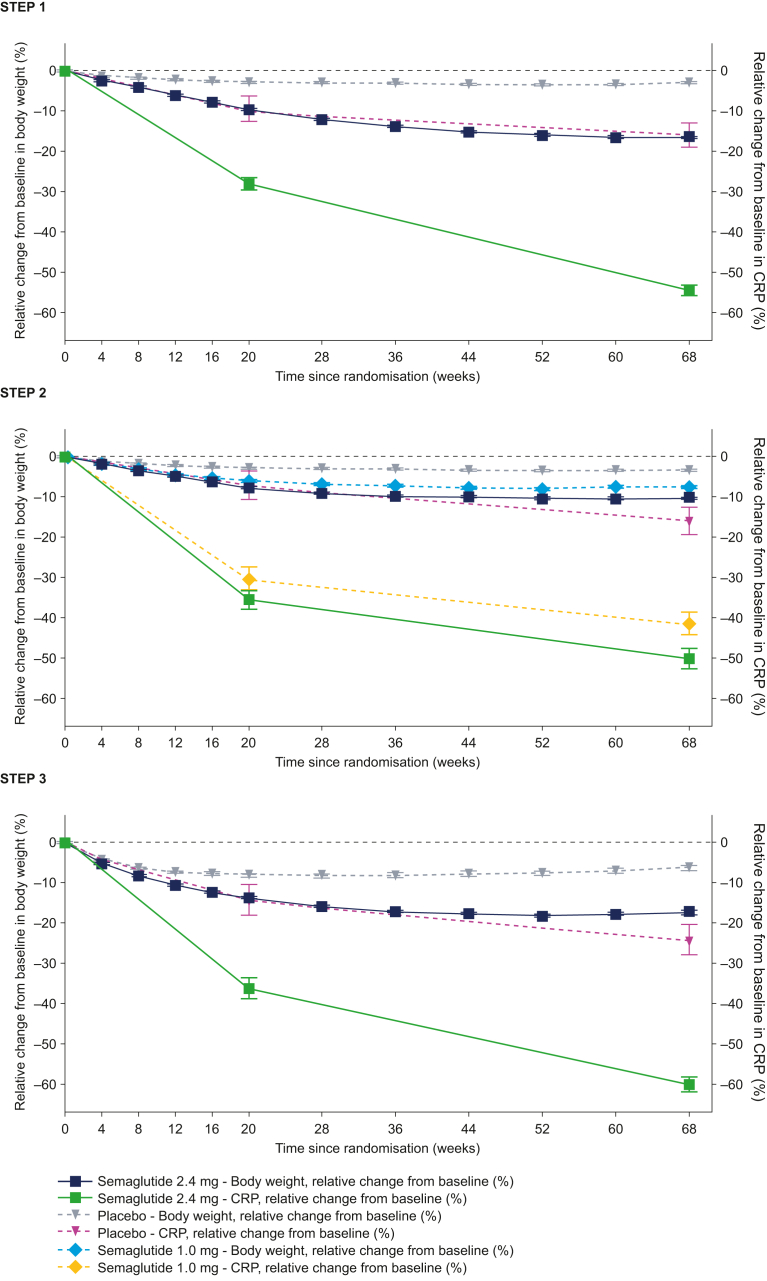
**Observed geometric mean change in CRP and observed mean change in bodyweight from baseline to week 68.** Observed data for the in-trial period (time from randomisation to last contact with the trial site, regardless of treatment discontinuation or rescue intervention) for the full analysis set. Error bars represent the standard error of the mean. Changes from baseline in CRP are based on observed ratios to baseline. They are presented as observed percent changes converted using the formula (observed ratio − 1) × 100. CRP, C-reactive protein. Novo Nordisk data published in refs.^13^, ^14^, ^15^

In all trials, CRP was reduced from baseline to week 68 with semaglutide 2.4 mg, 1.0 mg, and placebo (Fig. 1). The reductions with semaglutide 2.4 mg and 1.0 mg became apparent early (within the first 20 weeks) and occurred in parallel with bodyweight loss (Fig. 1). The reductions in CRP were significantly greater with semaglutide 2.4 mg versus placebo, with ETDs [95% CI] of −44% [–49 to −39] in STEP 1 (baseline CRP: 3.9 mg/L), −39% [–46 to −30] in STEP 2 (baseline CRP: 3.4 mg/L), and −48% [–55 to −39] in STEP 3 (baseline CRP: 4.5 mg/L; p < 0.001 for all), but not versus semaglutide 1.0 mg in STEP 2 (ETD: −12% [–23 to 1]; p = 0.0621; appendix p 3).

**Fig. 2:**
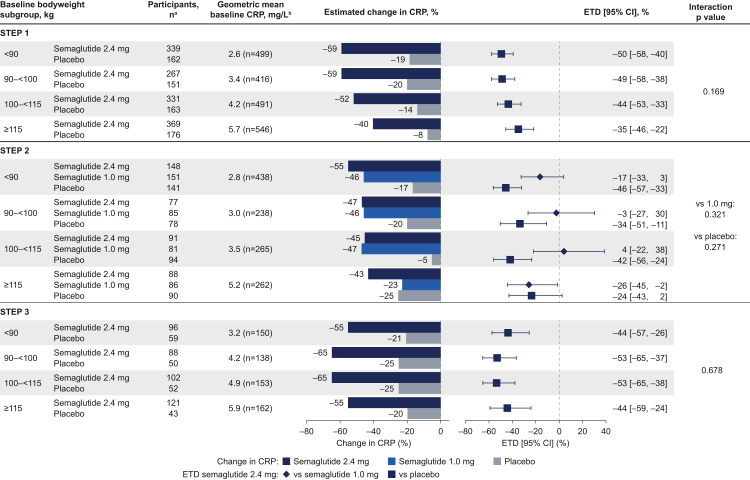
**Change in CRP from baseline to week 68 by baseline bodyweight.** Estimated data for the treatment policy estimand (assessed the treatment effect in all participants, regardless of trial product discontinuation or use of other anti-obesity therapies), analysed using the full analysis set. Changes from baseline and ETDs are based on estimated ratios to baseline and estimated treatment ratios. They are presented as estimated percent changes and estimated relative percent difference between groups, respectively, converted using the formula (estimated ratio − 1) × 100. CI, confidence interval; CRP, C-reactive protein; ETD, estimated treatment difference. ^a^Participant numbers represent the number of participants in the full analysis set (the number contributing to the analysis of estimated changes). ^b^Baseline values are observed data for the total population among participants with a baseline CRP assessment.

**Fig. 3:**
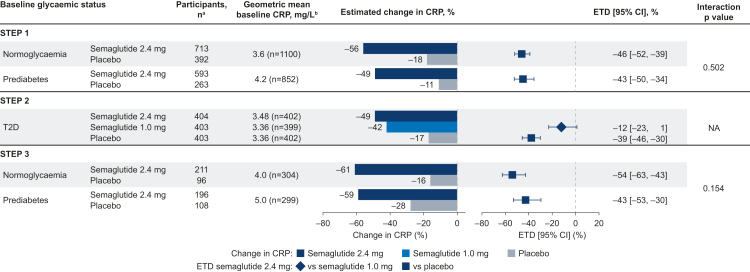
**Change in CRP from baseline to week 68 by baseline glycaemic status.** Estimated data for the treatment policy estimand (assessed the treatment effect in all participants, regardless of trial product discontinuation or use of other anti-obesity therapies), analysed using the full analysis set. Changes from baseline and ETDs are based on estimated ratios to baseline and estimated treatment ratios. They are presented as estimated percent changes and estimated relative percent difference between groups, respectively, converted using the formula (estimated ratio − 1) × 100. Data for the T2D population are based on the full analysis set in STEP 2 and therefore subgroup interactions were not assessed. CI, confidence interval; CRP, C-reactive protein; ETD, estimated treatment difference; NA, not assessed; T2D, type 2 diabetes. ^a^Participant numbers represent the number of participants in the full analysis set (the number contributing to the analysis of estimated changes). ^b^Baseline values are observed data for the total population among participants with a baseline CRP assessment.

There were greater reductions in CRP from baseline with semaglutide 2.4 mg versus placebo in all baseline bodyweight subgroups for all trials (Fig. 2). Baseline bodyweight subgroup did not affect the change in CRP with semaglutide 2.4 mg when compared with placebo or with semaglutide 1.0 mg, as there were no statistically significant interactions across these subgroups (all p > 0.05). Results were consistent across baseline BMI subgroups in all trials (data not shown) and among participants with prediabetes and normoglycaemia in STEP 1 and 3 (Fig. 3). Reductions in CRP were also similar in participants with type 2 diabetes (STEP 2) and participants with normoglycaemia and prediabetes (STEP 1 and 3; Fig. 3). When assessed on a continuous scale, the changes in CRP with semaglutide 2.4 mg, 1.0 mg, or placebo did not correlate with baseline bodyweight in any trial (appendix p 4).

Similarly, across all CRP subgroups, reductions in CRP from baseline were greater with semaglutide 2.4 mg versus placebo (all trials) and versus semaglutide 1.0 mg (STEP 2) across all baseline CRP subgroups (appendix p 5; all p values for interaction >0.05).

**Fig. 4:**
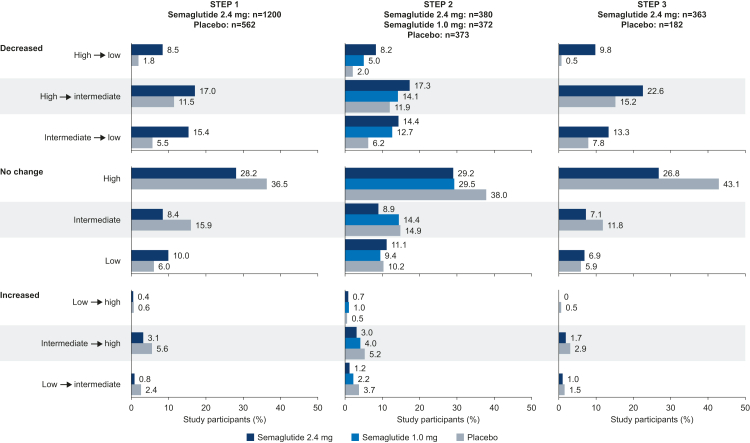
**Change in CRP-defined cardiovascular risk category from baseline to week 68.** Observed data for the in-trial period (time from randomisation to last contact with the trial site, regardless of treatment discontinuation or rescue intervention) for the full analysis set. Cardiovascular risk categories are defined as serum CRP levels of <1 mg/L (low), 1–3 mg/L (intermediate), and >3 mg/L (high). CRP, C-reactive protein.

Changes in CRP-defined cardiovascular risk were not analysed statistically, therefore, results are descriptive. In all trials, the proportion of participants with a reduction in CRP-defined cardiovascular risk appeared greater with semaglutide 2.4 mg versus placebo, and with semaglutide 1.0 mg versus placebo in STEP 2. Similar results, albeit to a lesser extent, were seen for semaglutide 2.4 mg versus 1.0 mg and for semaglutide 1.0 mg versus placebo in STEP 2 (Fig. 4). Fewer semaglutide-treated participants had an increase in cardiovascular risk compared with those receiving placebo in all trials (Fig. 4).

**Fig. 5:**
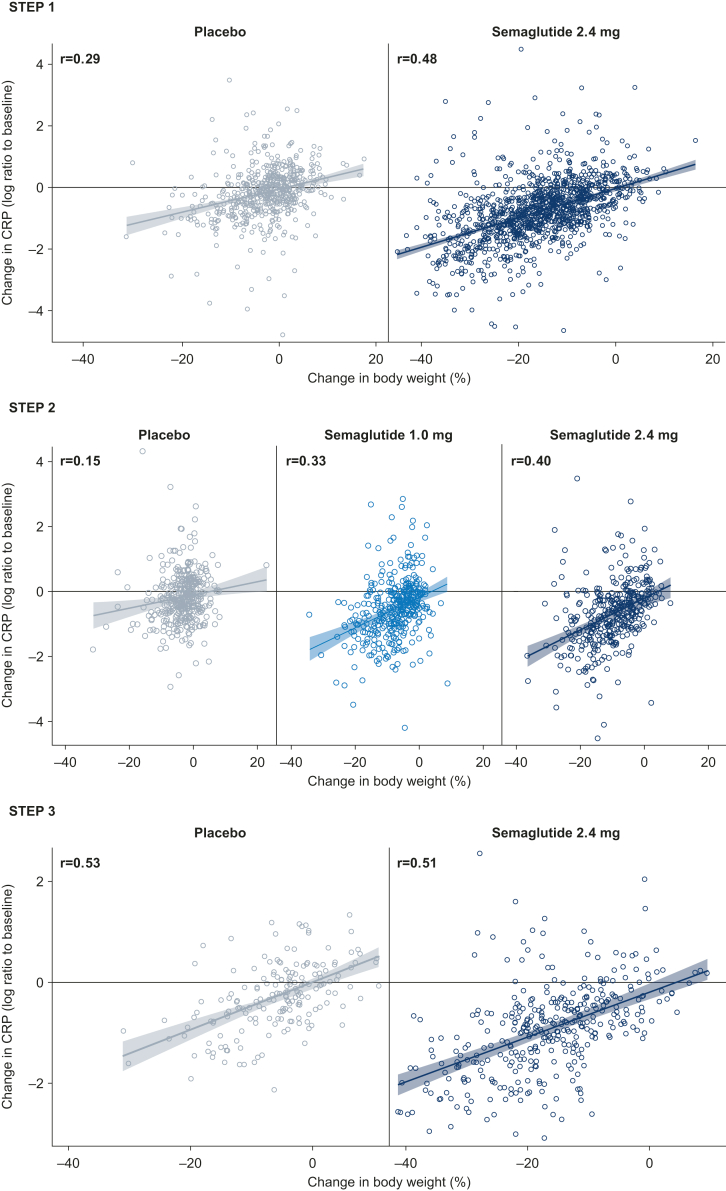
**Correlation between change in CRP and percent change in bodyweight from baseline to week 68.** Observed data for the in-trial period (time from randomisation to last contact with the trial site, regardless of treatment discontinuation or rescue intervention) for the full analysis set. CRP, C-reactive protein; r, Pearson correlation coefficient.

Change in CRP was positively correlated with changes in bodyweight, waist circumference, fasting plasma glucose, fasting serum insulin, and HOMA-IR, regardless of treatment allocation (fasting serum insulin was assessed in STEP 1 and 3 only) (Fig. 5; appendix pp 6–12). Overall, correlations were weak-to-moderate in strength, with Pearson correlation coefficients ranging from 0.14 to 0.51 with semaglutide 2.4 mg, 0.19 to 0.33 with semaglutide 1.0 mg, and 0.09 to 0.53 with placebo. In each trial, the strongest correlations observed were with change in bodyweight.

## Discussion

In the STEP 1, 2, and 3 trials in adults with overweight or obesity, with and without type 2 diabetes, treatment with semaglutide 2.4 mg resulted in significant reductions in CRP concentrations compared with placebo, irrespective of BMI or baseline bodyweight. These effects were also consistent across categories of glycaemic status and CRP at baseline. In the STEP 2 trial, there were non-significant reductions in CRP with semaglutide 2.4 mg compared with 1.0 mg. CRP reductions occurred in parallel with bodyweight loss and were positively correlated with reductions in bodyweight, waist circumference, fasting plasma glucose, fasting serum insulin, and HOMA-IR. In addition, greater proportions of participants treated with semaglutide had reductions in CRP-defined cardiovascular risk category compared with those on placebo.

The results of the present analyses are consistent with findings of a phase 2 trial with semaglutide at doses up to 0.4 mg/day (equivalent to 2.8 mg/week) in adults with obesity and without type 2 diabetes, which demonstrated significant, broadly dose-dependent reductions of CRP with semaglutide compared with placebo.^21^ After adjustment for bodyweight, the reductions in CRP were no longer statistically significant, suggesting a stronger influence of bodyweight loss on CRP reductions.^22^ However, such adjustments for post-randomisation variables are methodologically challenging, and disentangling the effects of semaglutide on CRP from those on weight loss remains difficult. Similar results have also been observed in people with type 2 diabetes; in the randomised, open-label, multinational 52-week PIONEER 2 trial, oral semaglutide 14 mg resulted in a significantly greater reduction in CRP compared with empagliflozin 25 mg.^23^ Liraglutide, a GLP-1 analogue closely related to semaglutide, has also been associated with significant reductions in CRP in people with obesity and/or type 2 diabetes.^10^ More broadly, a meta-analysis of trials investigating GLP-1RAs in people with type 2 diabetes demonstrated that GLP-1RA treatment was associated with significant reductions in CRP concentrations, with the duration of treatment associated with degree of CRP reduction.^9^ In our analysis of STEP 2, reductions in CRP were most pronounced up to week 20 but continued to fall after that time point.

It remains to be determined whether semaglutide has an effect on inflammation that is independent of (and additive to) weight loss in humans, although the evidence supporting this notion is growing and may, in part, be attributed to changes in vascular regenerative cell production.^24^, ^25^, ^26^ The present analysis was not designed to distinguish between the direct and indirect anti-inflammatory effects of semaglutide. It would be a significant challenge to separate these effects due to the low numbers of participants without substantial weight loss on semaglutide precluding a meaningful analysis.

In addition to being a biomarker of cardiovascular risk and proposed clinical marker to guide treatment for cardiovascular disease events,^2^^,^^3^ elevated CRP is a significant predictor for future diagnosis of metabolic syndrome and type 2 diabetes.^2^ In this analysis, CRP reduction was significantly greater with semaglutide compared with placebo in the STEP 1 and 3 trials of people with overweight or obesity without diabetes. Furthermore, these reductions were correlated with reductions in fasting serum insulin and HOMA-IR. This supports the notion that reducing inflammatory processes, as measured by CRP, in addition to lowering insulin levels and improving insulin sensitivity, could contribute to protective effects against development of type 2 diabetes and progression of cardiometabolic disease.^27^^,^^28^

People with type 2 diabetes are at increased risk of cardiovascular events.^9^^,^^29^ Inflammation, as reflected by elevated serum CRP, also appears to be involved in cardiovascular morbidity and mortality in people with metabolic syndrome and type 2 diabetes.^2^ In this analysis of the STEP 2 trial in people with type 2 diabetes, CRP levels were reduced with both semaglutide 2.4 mg and 1.0 mg versus placebo, with no significant difference between the semaglutide doses. Furthermore, correlations between changes in CRP concentration and other cardiometabolic outcomes were similar in strength for semaglutide 2.4 mg and 1.0 mg for all endpoints assessed. This suggests that reductions in CRP may also be associated with improvements in other cardiovascular risk factors in people with type 2 diabetes. In SUSTAIN 6, semaglutide significantly reduced the risk of major adverse cardiovascular events compared with placebo, and was non-inferior to placebo in PIONEER 6, in people of varying cardiovascular risk, with type 2 diabetes.^30^^,^^31^ However, caution must be used comparing these trials to the present analysis due to differences in trial design and patient populations.

Semaglutide is indicated to reduce the risk of major adverse cardiovascular events in adults with type 2 diabetes and established cardiovascular disease at the dose of 1.0 mg once weekly.^11^ The ongoing Semaglutide Effects on Heart Disease and Stroke in Patients with Overweight or Obesity trial will enrol 17 500 participants and investigate whether semaglutide 2.4 mg is superior to placebo for preventing major adverse cardiovascular events in participants with established cardiovascular disease and overweight or obesity, without diabetes.^32^ It will not, however, assess the possible direct and indirect effects of semaglutide.

There are several limitations of these analyses. As they were exploratory in nature and conducted *post hoc*, they were not adjusted for multiple comparisons. Exploratory subgroup analyses are subject to false positive, or negative, findings and so caution should be taken when interpreting the results.^33^ The STEP trials did not measure other markers of systemic inflammation, such as interleukin-6 or tumour necrosis factor alpha, that could have provided greater insight. This analysis did not investigate what effects the reductions in CRP might have had on the number of subsequent cardiovascular events among participants of the STEP 1, 2, and 3 trials. To this end, it is important to note that CRP is a marker of inflammation and cardiovascular risk that does not directly lead to adverse cardiovascular outcomes. CRP is closely linked to multiple cardiovascular risk factors (as well as adiposity) and therefore the relationship between CRP and cardiovascular outcome may reflect changes other than weight status or even the direct effects of semaglutide on atherosclerosis. These points should be considered when interpreting the results of this study. Furthermore, this analysis only provides data for 68 weeks of semaglutide treatment; analysis of the changes in CRP beyond this time period may be beneficial to explore the effects of semaglutide treatment over the longer-term.

In conclusion, treatment with once-weekly s.c. semaglutide 2.4 mg compared with placebo led to substantial reductions in body weight and reduced CRP concentration in people with overweight or obesity, with or without type 2 diabetes. Reductions in CRP concentration were consistent across baseline BMI, bodyweight, glycaemic status, and CRP levels. These results indicate that the beneficial weight-lowering effects of semaglutide treatment are associated with improvements in systemic inflammation in these patient populations.

## Contributors

SV, MB, JED, CJ, KK, RFK, and MNK contributed to data collection, analysis, and interpretation, and manuscript development. MD and DMR contributed to the conduct of the trial, data collection, analysis, and interpretation, and manuscript development. TWG contributed to the conduct of the trial, data collection, interpretation, and manuscript development. All authors had full access to all the data in the study and had final responsibility for the decision to submit for publication. All authors contributed to the data interpretation and manuscript writing (assisted by a medical writer paid for by the funder), approved the final version of the manuscript, and vouch for data accuracy and fidelity to the protocol.

## Data sharing statement

Data will be shared with bona fide researchers who submit a research proposal approved by the independent review board. Individual patient data will be shared in data sets in a de-identified and anonymised format. Data will be made available after research completion and approval of the product and product use in the EU and the USA. Information about data access request proposals can be found at novonordisk-trials.com.

## Declaration of interests

Dr Bhatta is an employee of Novo Nordisk A/S.

Professor Davies reported receiving research funding from AstraZeneca, Boehringer Ingelheim, Janssen, 10.13039/501100004191Novo Nordisk, and Sanofi-Aventis; has acted as consultant, advisory board member, and speaker for Boehringer Ingelheim, Eli Lilly, Novo Nordisk, Sanofi-Aventis; advisory board member and speaker for AstraZeneca; advisory board member for Gilead Sciences Ltd, Janssen, and Lexicon; and speaker for Napp Pharmaceuticals and Takeda Pharmaceuticals International Inc. She is co-funded by the NIHR Leicester Biomedical Research Centre.

Dr. Deanfield reports personal fees from Amgen, Boehringer Ingelheim, Merck, Pfizer, Aegerion, Novartis, Sanofi, Takeda, Novo Nordisk, Bayer, grants from BHF, MRC(UK), NIHR, Public Health England, MSD, Pfizer, Cancer Research UK, Alzheimer’s Research UK, other from Novo Nordisk, outside the submitted work.

Dr. Garvey reports grants from 10.13039/501100004191Novo Nordisk, during the conduct of the study; grants from Novo Nordisk, grants from Eli Lilly, grants from Epitomee, grants from Pfizer, personal fees from Boehringer Ingelheim, personal fees from Novo Nordisk, personal fees from Fractyl Health, personal fees from Alnylam Pharmaceuticals, personal fees from Merck, personal fees from Eli Lilly, outside the submitted work.

Camilla Jensen is an employee of Novo Nordisk A/S.

Dr Kandler is an employee of Novo Nordisk A/S.

Dr. Kosiborod reports grants, personal fees and other from AstraZeneca, personal fees from Alnylam, personal fees from Amgen, personal fees from Applied Therapeutics, personal fees from Bayer, grants and personal fees from Boehringer Ingelheim, personal fees from Cytokinetics, personal fees from Eli Lilly, personal fees from Esperion Therapeutics, personal fees from Janssen, personal fees from Lexicon, personal fees from Merck (Diabetes and Cardiovascular), personal fees from Novo Nordisk, personal fees from Pharmacosmos, personal fees from Sanofi, personal fees from Vifor Pharma, outside the submitted work.

Dr. Kushner reports personal fees from Novo Nordisk, personal fees from Eli Lilly, outside the submitted work.

Dr. Rubino reports other from Novo Nordisk, during the conduct of the study; personal fees and other from Novo Nordisk, personal fees and other from Boehringer Ingelheim, personal fees from Endocrine Society, PeerView, WebMD, outside the submitted work.

Dr. Verma reports grants and personal fees from Amarin, grants and personal fees from Amgen, grants and personal fees from Bayer, grants and personal fees from Boehringer Ingelheim, personal fees from Canadian Medical and Surgical Knowledge Translation Research Group, grants and personal fees from Eli Lilly, personal fees from EOCI, grants and personal fees from HLS Therapeutics, personal fees from Janssen, personal fees from Novartis, grants and personal fees from Novo Nordisk, personal fees from Otsuka, grants and personal fees from Pfizer, grants and personal fees from PhaseBio, personal fees from Sanofi, personal fees from Sun Pharma, personal fees from TKTWG, outside the submitted work.
