# Supplementary figures and images for: Effects of once-weekly semaglutide 2.4 mg on C-reactive protein in adults with overweight or obesity (STEP 1, 2, and 3): Exploratory analyses of three randomised, double-blind, placebo-controlled, phase 3 trials
Source: eClinicalMedicine. 2022 Nov 29;55:101737. doi: 10.1016/j.eclinm.2022.101737 (PMC9713290; doi:10.1016/j.eclinm.2022.101737)

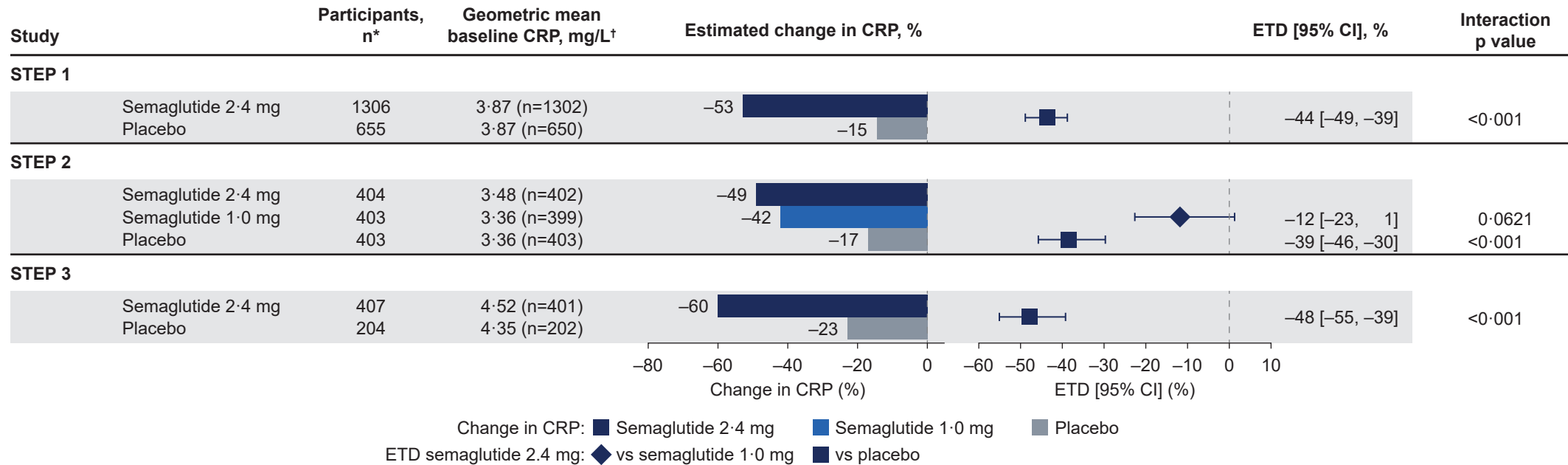

Supplement: Supplementary Fig. S1 [file mmc1.pdf]

STEP 1

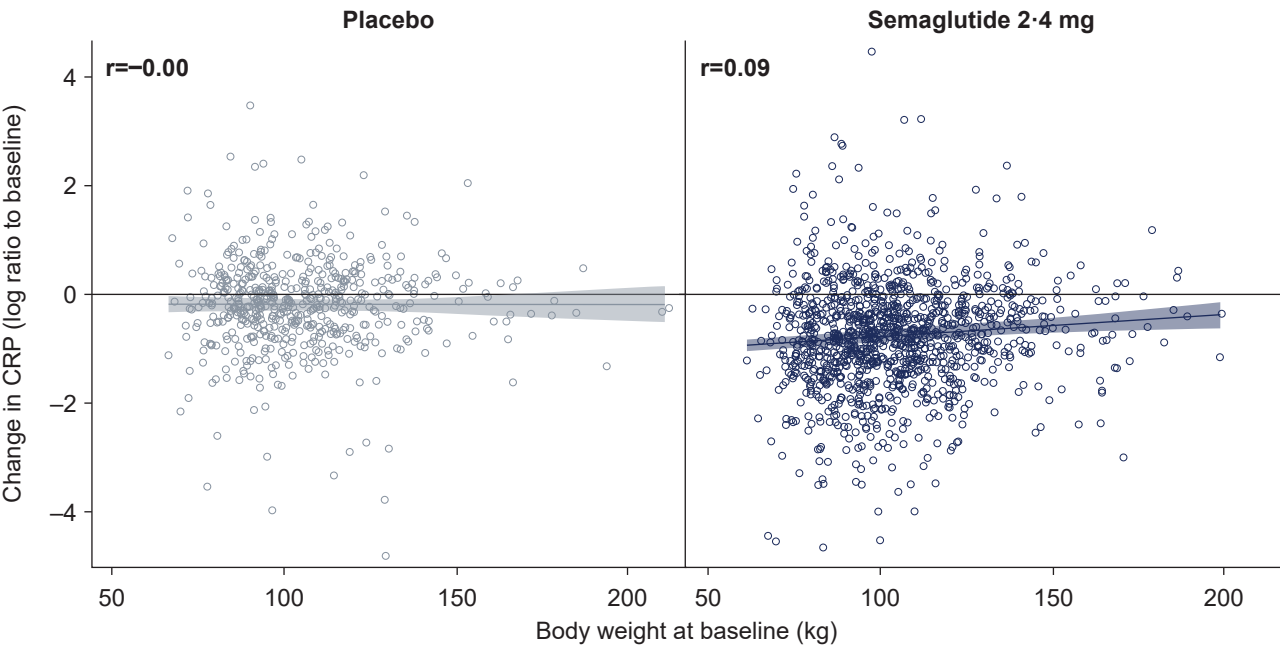

STEP 2

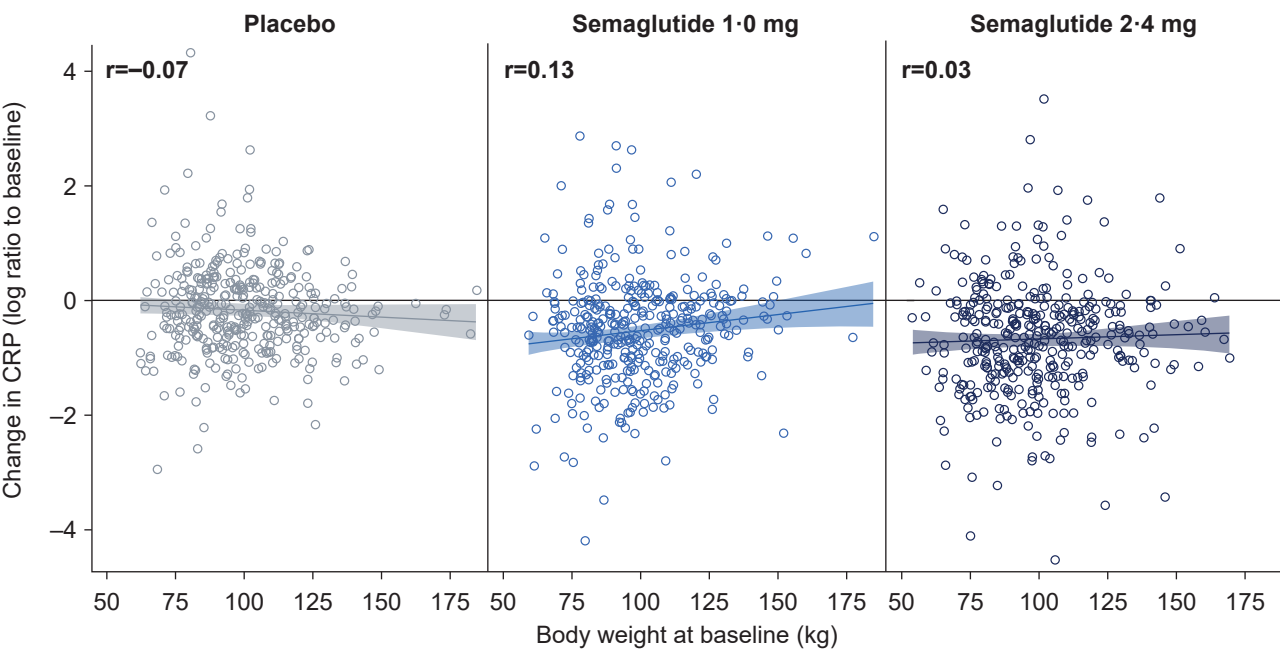

STEP 3

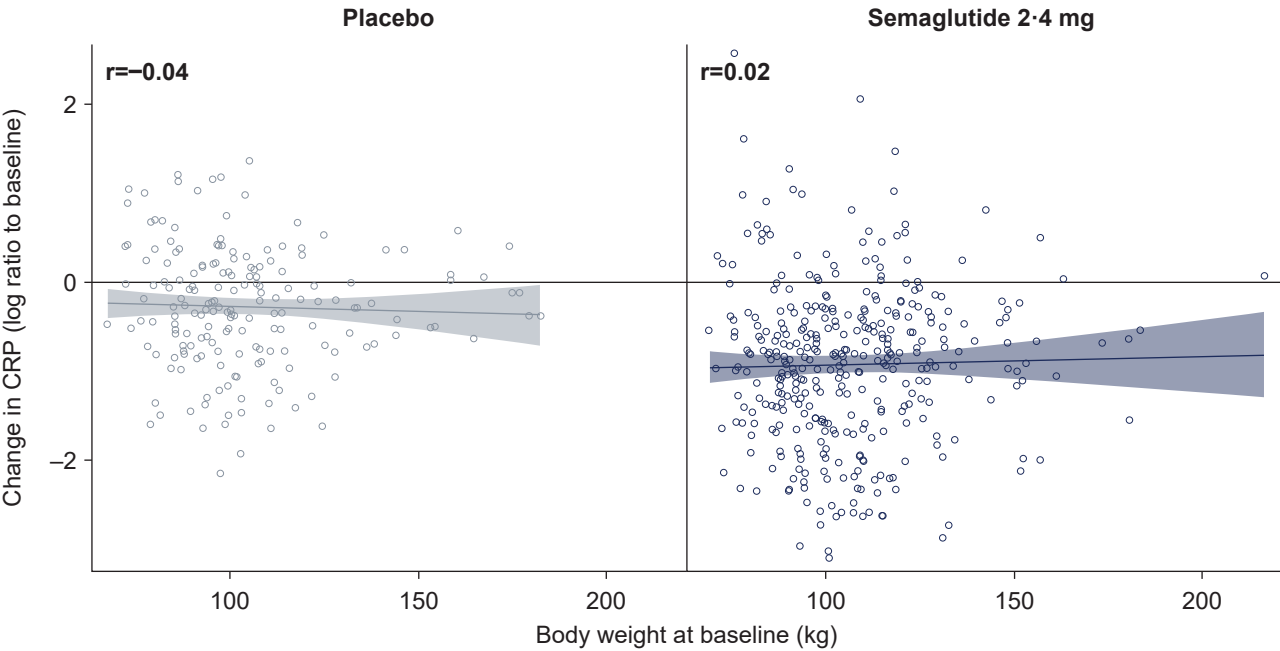

Supplement: Supplementary Fig. S2 [file mmc2.pdf]

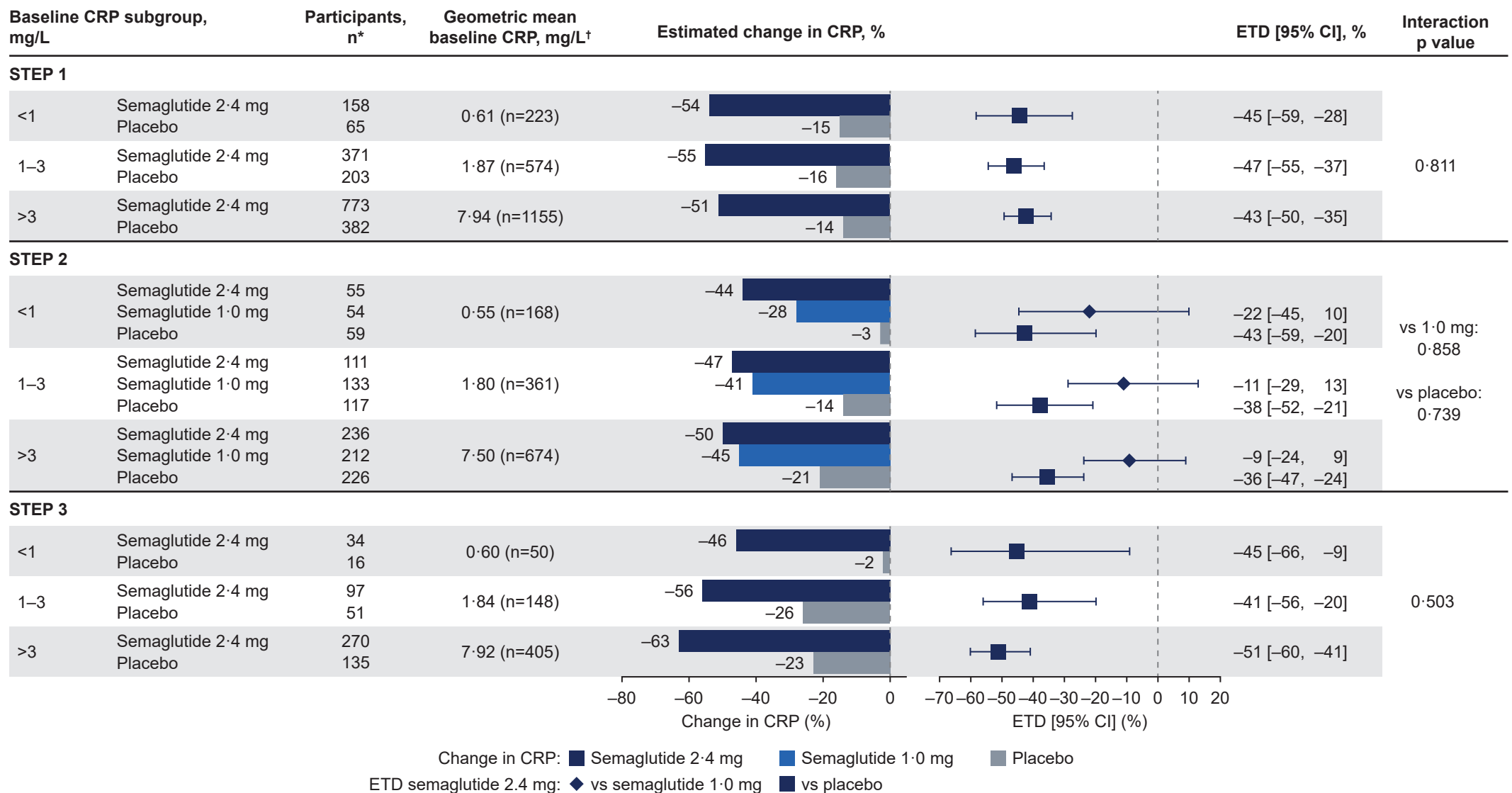

Supplement: Supplementary Fig. S3 [file mmc3.pdf]

STEP 1

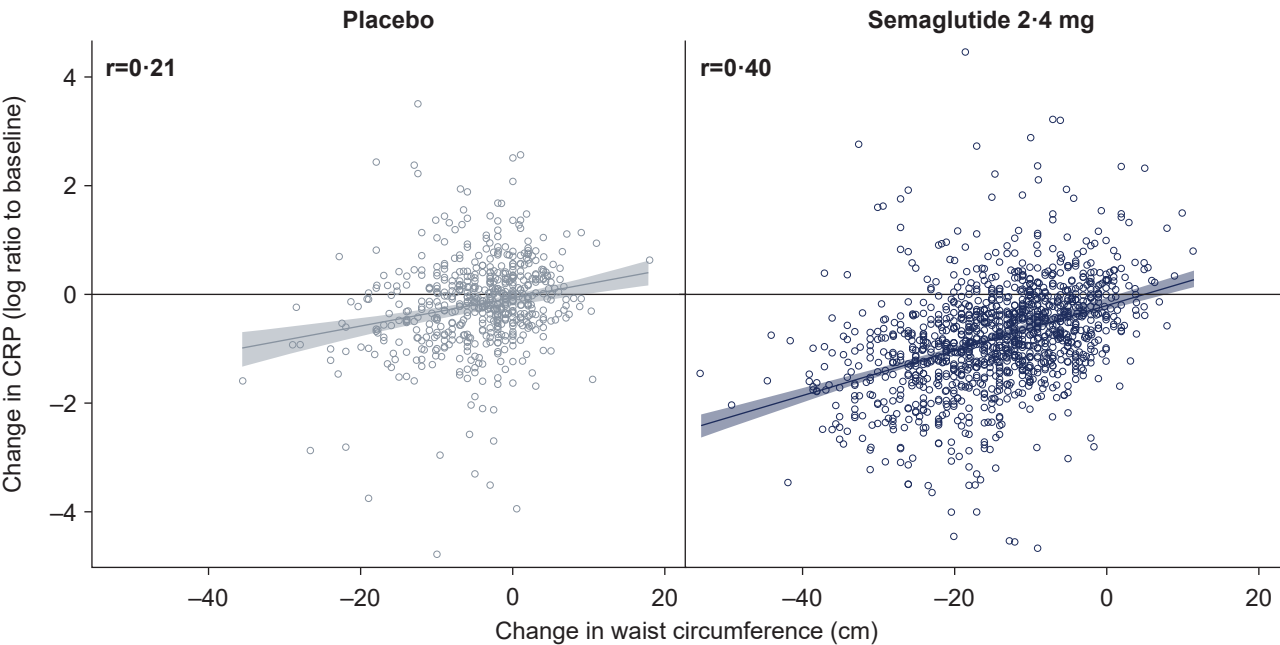

STEP 2

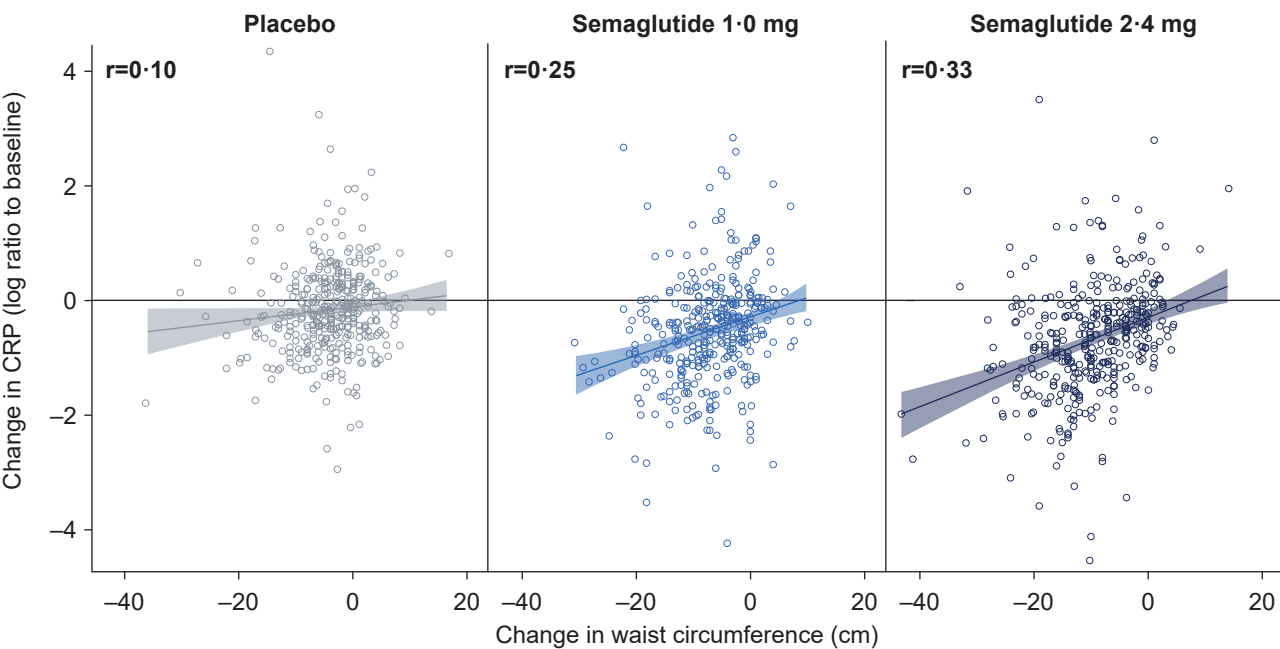

STEP 3

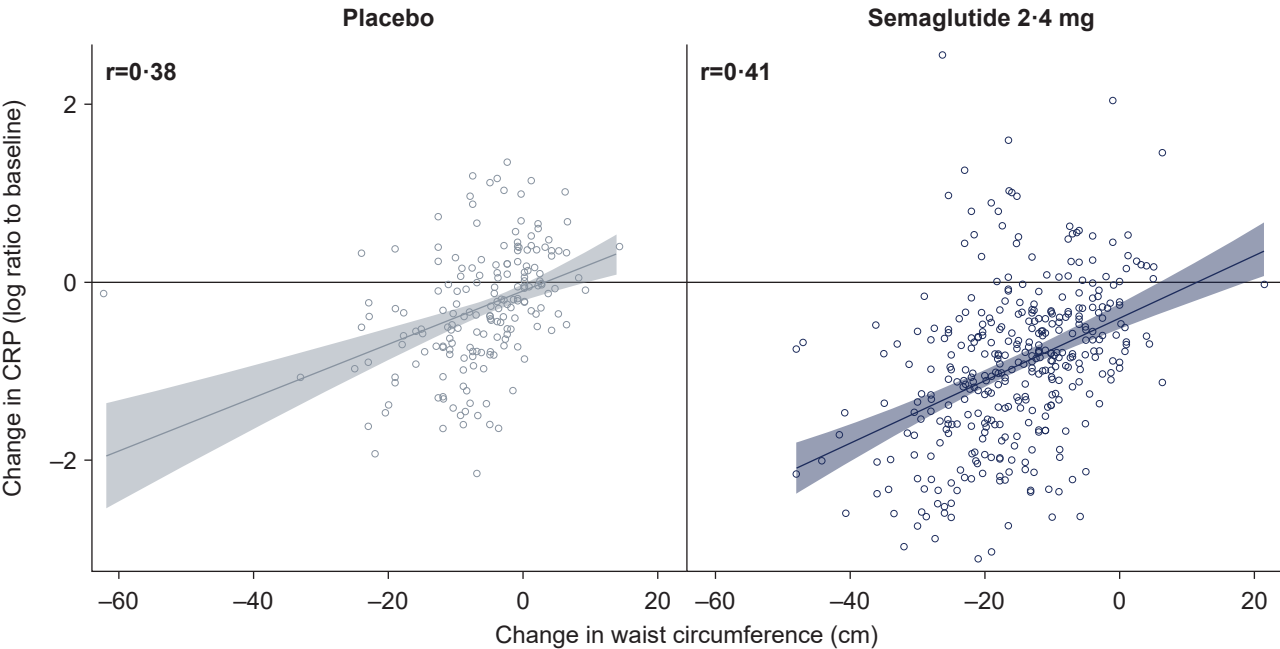

Supplement: Supplementary Fig. S4 [file mmc4.pdf]

STEP 1

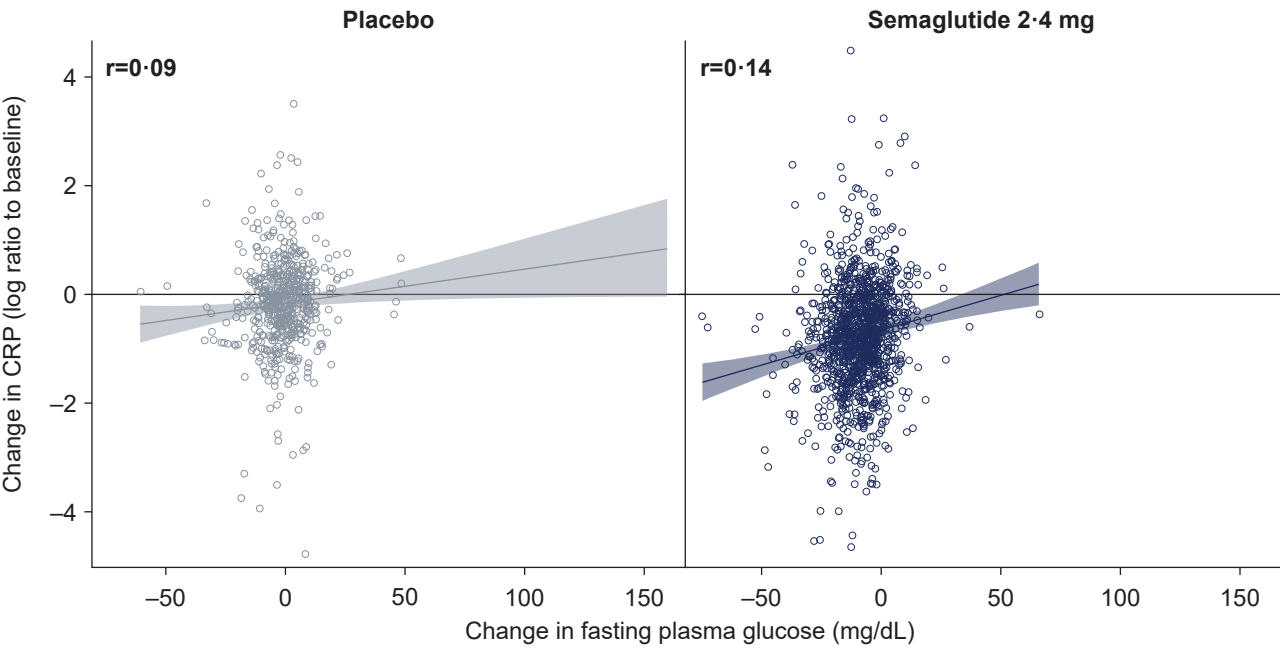

STEP 2

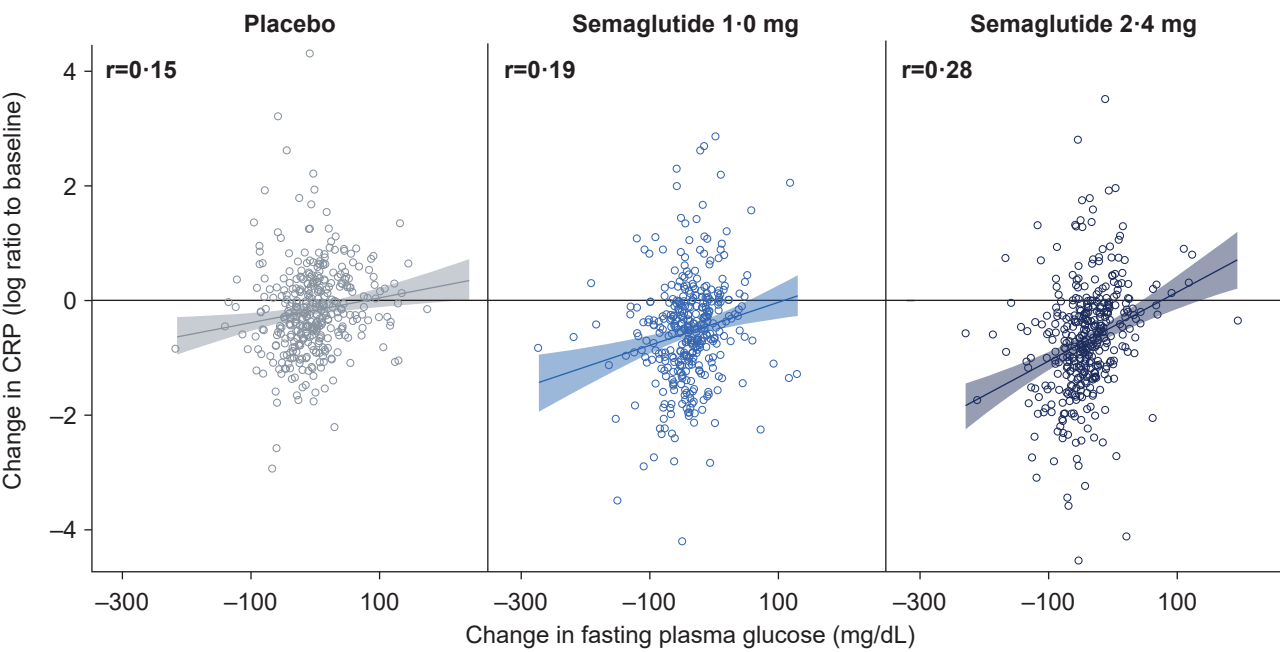

STEP 3

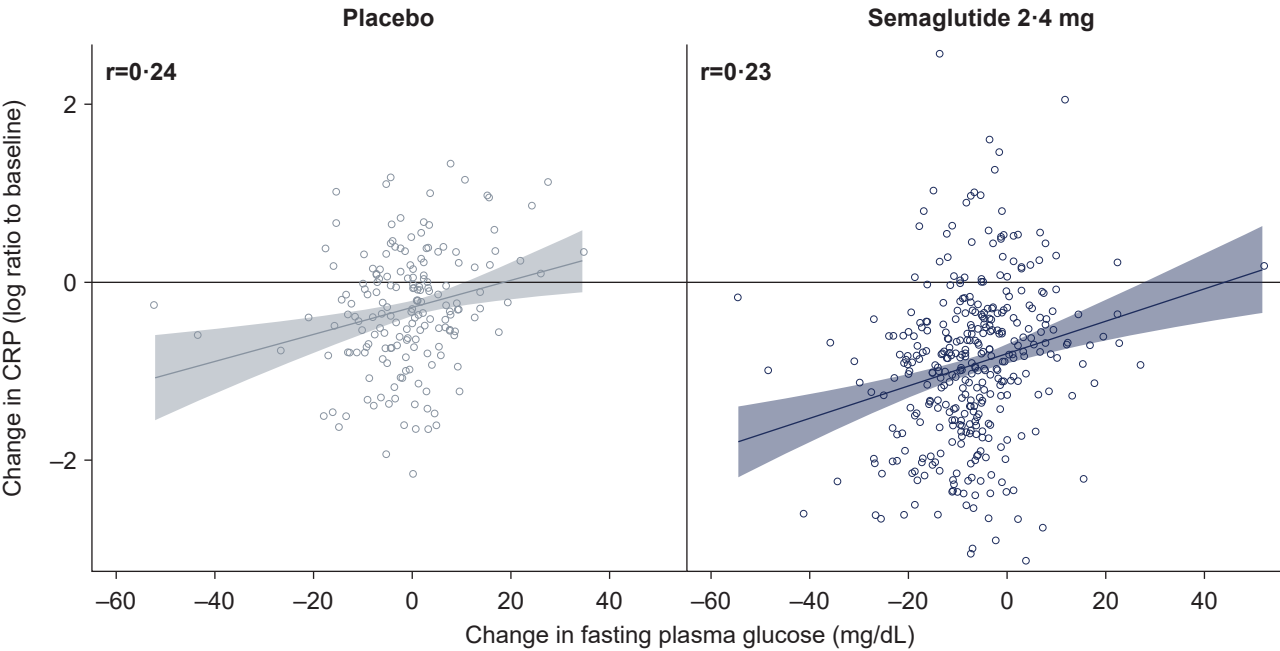

Supplement: Supplementary Fig. S5 [file mmc5.pdf]

**STEP 1**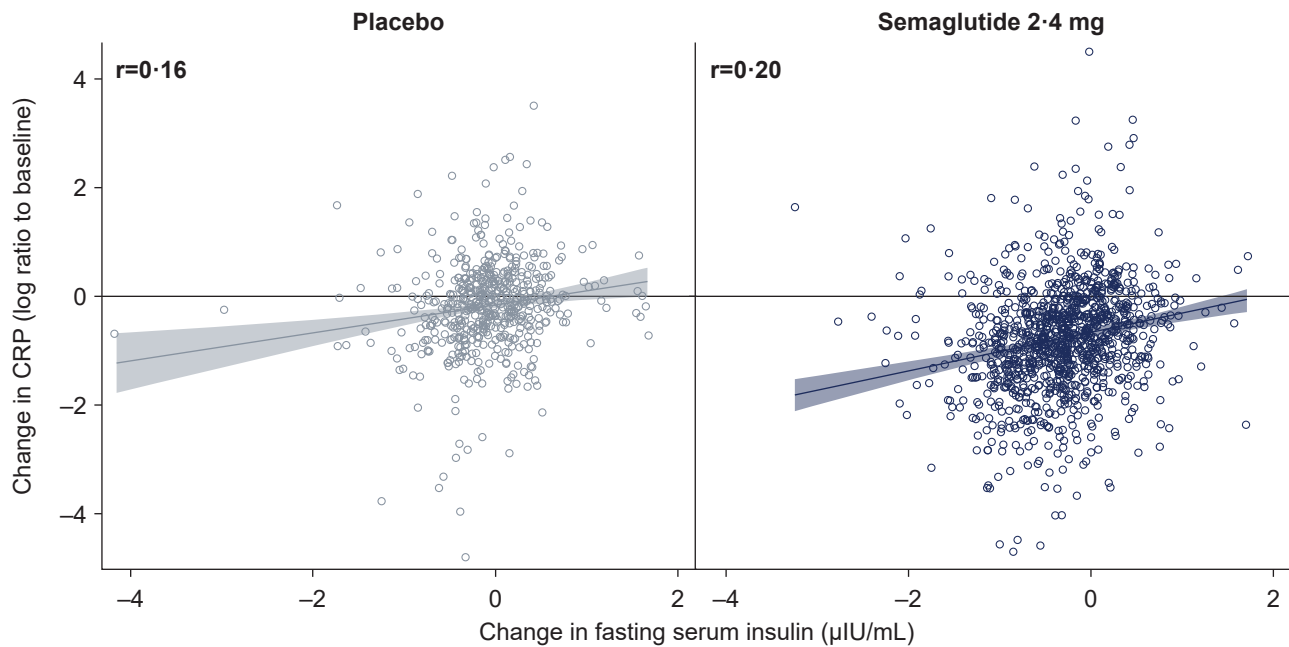**STEP 3**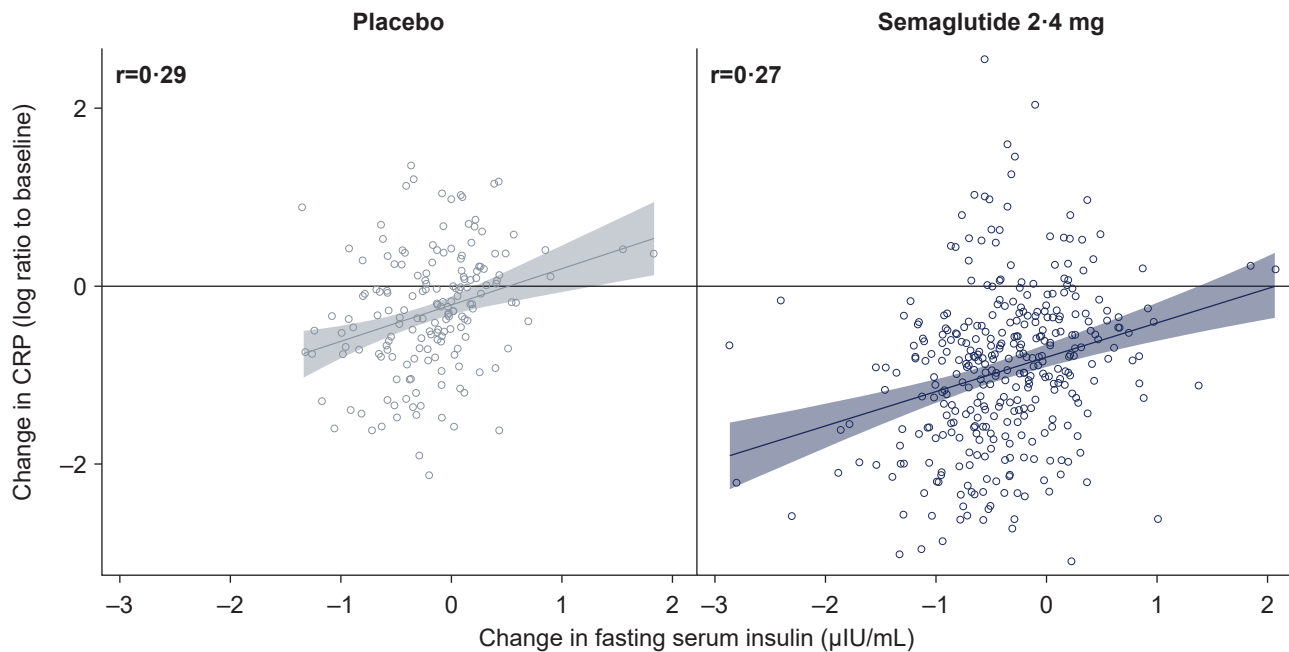

Supplement: Supplementary Fig. S6 [file mmc6.pdf]

STEP 1

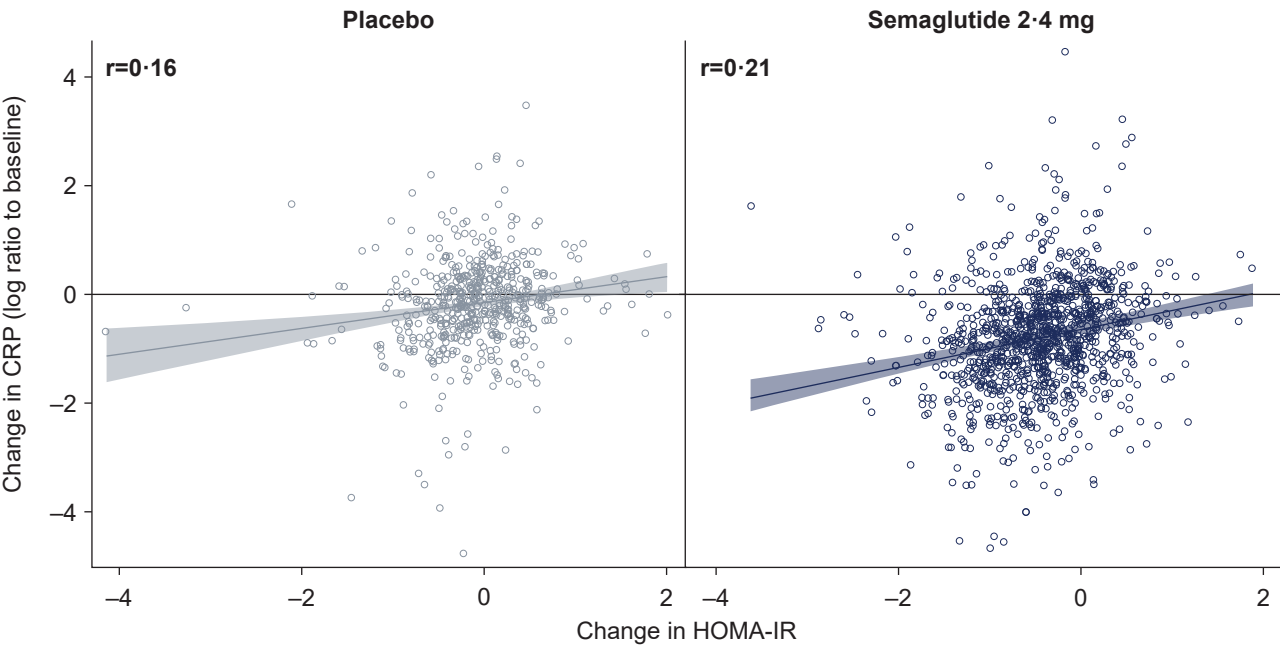

STEP 2

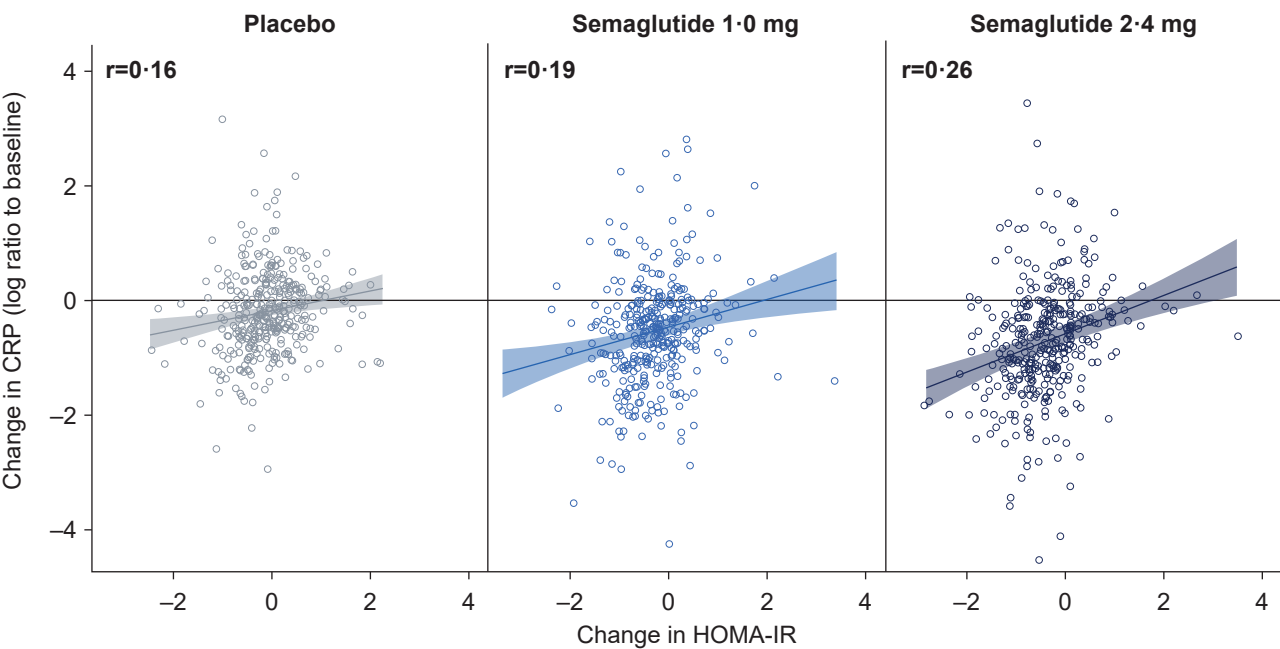

STEP 3

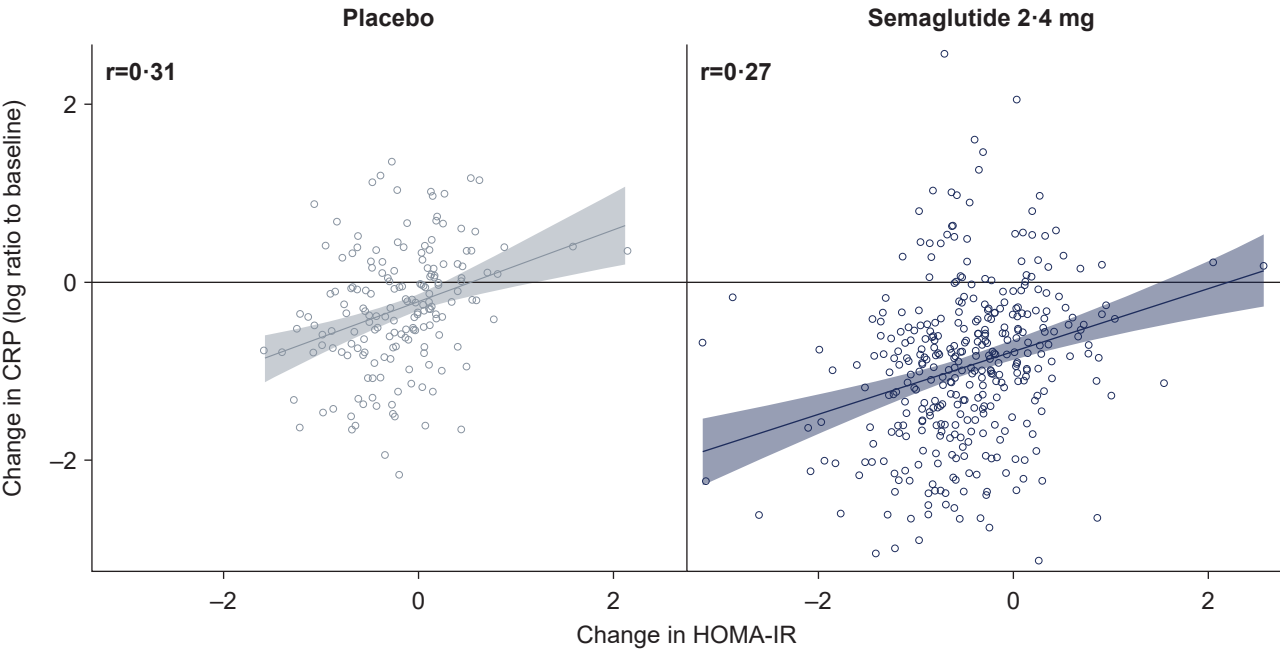

Supplement: Supplementary Fig. S7 [file mmc7.pdf]
