# Supplementary material for: Effects of once-weekly semaglutide 2.4 mg on C-reactive protein in adults with overweight or obesity (STEP 1, 2, and 3): Exploratory analyses of three randomised, double-blind, placebo-controlled, phase 3 trials
Source: eClinicalMedicine. 2022 Nov 29;55:101737. doi: 10.1016/j.eclinm.2022.101737 (PMC9713290; doi:10.1016/j.eclinm.2022.101737)
Supplement: Supplementary File S1 [file mmc8.docx]

STEP 1-3 CRP manuscript supplementary material
